# Determining the Peak Power Output for Weightlifting Derivatives Using Body Mass Percentage: A Practical Approach

**DOI:** 10.3389/fspor.2021.628068

**Published:** 2021-04-20

**Authors:** Marcel Lopes dos Santos, Adam Jagodinsky, Kristen M. Lagally, Valmor Tricoli, Ricardo Berton

**Affiliations:** ^1^School of Kinesiology and Recreation, Illinois State University, Normal, IL, United States; ^2^School of Kinesiology, Applied Health and Recreation, Oklahoma State University, Stillwater, OK, United States; ^3^School of Physical Education and Sport, University of São Paulo, São Paulo, Brazil

**Keywords:** Olympic weightlifting, weightlifting pulling derivatives, power training, optimal load, sport performance

## Introduction

Mechanical power is a key component for sports performance (Kawamori and Haff, 2004; Haff and Nimphius, 2012). Several studies have demonstrated moderate to strong associations between mechanical power and important sports tasks, such as vertical jump and sprint performance (Loturco et al., 2015a,c; Marcote-Pequeño et al., 2019). For this reason, strength and conditioning coaches dedicate a significant amount of attention to training strategies aimed at increasing athletes' mechanical power capability. One of the appropriate strategies to accomplish this goal is to perform a given exercise at the load that acutely results in the highest peak power output (PPO), described by many authors as the “optimal load” (Kaneko et al., 1983; Wilson et al., 1993; Kawamori and Haff, 2004; Cormie et al., 2011; Loturco et al., 2016; Sarabia et al., 2017). Thereby, numerous researchers have investigated the PPO for exercises frequently used in physical training programs (Cormie et al., 2007c; McBride et al., 2011; Comfort et al., 2012a; Suchomel and Sole, 2017).

Weightlifting derivatives (i.e., exercises that omit a portion of the snatch or clean and jerk) (Suchomel et al., 2018) are regularly used in power training programs (Ebben and Blackard, 2001; Durell et al., 2003; Ebben et al., 2004; Simenz et al., 2005; Gee et al., 2011; Jones et al., 2016). Consequently, the PPO for these exercises have been studied extensively. For example, the PPO of the jump shrug (JShrug) (Suchomel et al., 2014b, 2016; Suchomel and Sole, 2017; Kipp et al., 2018), countermovement shrug (CShrug) (Meechan et al., 2020b), hang high pull (HHP) (Suchomel et al., 2014b, 2015a; Suchomel and Sole, 2017), hang clean pull (HCP) (Meechan et al., 2020a), mid-thigh clean pull (MTCP) (Haff et al., 1997; Kawamori et al., 2006; Comfort et al., 2012b, 2015), hang power clean (HPC) (Kawamori et al., 2005; Kilduff et al., 2007; Suchomel et al., 2014a,b; Suchomel and Sole, 2017; Kipp et al., 2018), and power clean (PC) (Cormie et al., 2007a,b,c; McBride et al., 2011; Comfort et al., 2012a) have been previously identified. Despite the importance of prescribing exercises at the load at which the PPO is achieved, the means by which PPO values are obtained may present as a barrier to practitioners. Usually, PPO is represented by a relative percentage of the one-repetition maximum test (1RM) (Kawamori et al., 2006; Cormie et al., 2007a,c; Kilduff et al., 2007; McBride et al., 2011; Comfort et al., 2012b, 2015; Suchomel et al., 2014a,b; Suchomel et al., 2015a, 2016; Kipp et al., 2018). Although this strategy is effective, 1RM testing in weightlifting derivatives presents some challenges. Specifically, 1RM testing can be very time-consuming (Chapman et al., 1998; Niewiadomski et al., 2008; Loturco et al., 2017a), and also be impractical for some weightlifting derivatives (e.g., JShurg, HHP, and MTCP) (Suchomel and Sole, 2017; Suchomel et al., 2019). Moreover, because there are no standard 1RM test procedures for some of them, relative loads from different exercises (HPC and PC) are used as a reference for load attainment. These disadvantages will be further addressed in the next section.

Given the disadvantages of using the 1RM test, it has become apparent that there is a need for practical alternatives to determine the PPO in the weightlifting derivatives. A practical alternative that emerges as a possible solution is the use of a relative percentage based on the body mass (BM) of the athletes (Suchomel et al., 2019). The use of this alternative may offer advantages as it eliminates the need to perform 1RM tests and to prescribe loads based on different exercises (Suchomel et al., 2019). Therefore, the purpose of the manuscript is to provide a rationale for the use of BM as an alternative for PPO identification and also to explore advantages and disadvantages of this method.

## PPO Based on the Relative Percentage of 1RM

As mentioned in the previous section, the PPO is typically represented by a percentage value of the 1RM test (Kawamori et al., 2006; Cormie et al., 2007a,c; Kilduff et al., 2007; McBride et al., 2011; Comfort et al., 2012b, 2015; Suchomel et al., 2014a,b, 2015a, 2016; Kipp et al., 2018). Thus, the 1RM test can be considered a relevant assessment for training prescription. However, although effective, the 1RM test may be impracticable in athletic settings. In large rosters of athletes, the application of 1RM tests for a single exercise can take hours depending on the personnel involved and number of available stations (Chapman et al., 1998; Niewiadomski et al., 2008; Loturco et al., 2017a). Thereby, the 1RM test may be impractical or at least very time consuming/labor-intensive (Loturco et al., 2017a) for strength and conditioning coaches that have a limited time to develop and implement their training programs. This directly affects professionals working with collegiate athletes, once the NCAA enforces a maximum of 20 h/week during the in-season dedicated to athletically related activities and only 8 h/week during the offseason (National Collegiate Athletic Association, 2009).

Another disadvantage of the 1RM test that can be specifically observed in the weightlifting derivatives is the conduction of 1RM tests in exercises different from those undertaken during the training program. For weightlifting pulling derivatives (WPD) (i.e., exercises without the catch phase—JShrug, HHP, and MTCP), 1RM tests are not feasible because it is difficult to set specific criteria for what may be considered a successful repetition and/or a complete movement cycle (Suchomel and Sole, 2017; Suchomel et al., 2019). For this reason, PPO for the JShrug, HHP, and MTCP has been represented by percentage loads of 1RM of weightlifting catching derivatives (WCD) (i.e., exercises with the catch phase— PC or HPC) (Kawamori et al., 2006; Comfort et al., 2012b, 2015; Suchomel et al., 2014b, 2015b, 2016; Kipp et al., 2018). This practical solution for PPO prescription can be considered disadvantageous. When WPD are employed in training programs (i.e., exercises of less technical complexity) (Suchomel et al., 2014b, 2015b; Suchomel and Sole, 2017), the athletes are still required to learn and be proficient in WCD (i.e., exercises of high technical complexity) only for load prescription (Comfort et al., 2018; Suchomel et al., 2020). In other words, even when utilizing exercises with lesser technical complexity and; therefore, easier to learn and implement in training programs, athletes are still required to dedicate a significant amount of time to the learning of highly complex exercises. As previously mentioned, strength and conditioning coaches may have limited time to work on athletically related activities with their athletes. In that case, time allocated to learning the WCD would take away from (or minimize) time that could be dedicated to other practices and training-related activities.

It is also important to note that even in situations in which time is not an issue, the use of WCD may not be the most advantageous option. In general, WCD produce similar or inferior power output in comparison to WPD (Comfort et al., 2011; Suchomel et al., 2014b; Suchomel and Sole, 2017). From this perspective, when the aim is to maximize power output in a specific time period, WPD offer an appropriate stimulus while being less technically complex than WCD (Suchomel et al., 2015b). Thus, exercises such as JShrug, HHP, and MTCP may be considered advantageous options in training programs. As such, finding an alternative for PPO identification in WPD will benefit athletes and strength and conditioning coaches.

## PPO Based on the Relative Percentage of BM

An alternative that may facilitate the prescription of the PPO for the WPD is the use of BM. From a practical standpoint, the athlete can perform repetitions with progressive loads, for example, starting at 20% of their BM with subsequent increments of 10–20% until the individual PPO at a given exercise is achieved (Loturco et al., 2015b; Suchomel et al., 2019). Indeed, the BM has been used as a parameter for neuromuscular assessments mainly in exercises such as jump squat and bench press (Loturco et al., 2015b, 2017b; Rauch et al., 2018). On the other hand, the use of BM to identify PPO in WPD is still scarce.

To date, only one study used BM to identify PPO in WPD. Suchomel et al. (2019) identified the PPO based on a relative percentage of BM for the JShrug. Each participant performed repetitions of the JShrug from 0% (only a PVC pipe) to 100% of their BM. The PPO was found at 20% BM, although no statistical difference was observed compared to 0, 40, and 60% BM. From their findings, it is plausible to suggest that this alternative offers an advantage for PPO determination (Suchomel et al., 2019). As the only parameter required for the determination of the training load is the participants' BM, this strategy mitigates the need for and consequently, the disadvantages of the 1RM test. As such, strength and conditioning coaches are encouraged to use the body mass procedure in other WPD.

Despite the advantages, BM also presents limitations. Athletes with greater maximal strength levels are expected to reach the PPO at greater relative loads than weaker athletes (Stone et al., 2003). From a practical standpoint, two athletes with different maximal strength levels may achieve PPO at different percentages of their BM. For this reason, the BM percentage at which the PPO was found cannot be extrapolated from one athlete to another. Therefore, the prescription of the optimal load based on BM should be made with caution and performed through individual tests (e.g., force platform or linear position transducers) when the aim is to improve the PPO of the system (individual's BM + external load) or barbell, respectively (Soriano et al., 2020). It should be mentioned that although devices for data collection are necessary, these are increasingly portable, low cost, and; therefore, very accessible to strength and conditioning coaches. Another limitation is related to athletes' changes in BM throughout the season. To date, no study has investigated whether the change in the BM may affect the determination of the PPO. Thus, as a precaution, we recommend PPO prescription based on BM only for athletes who portray minor BM variation throughout the season (French et al., 2004; Clark et al., 2008). Finally, despite the limitations, the proposal described in this manuscript should not be discarded. When performing individual tests, the use of BM may be a valuable tool for strength and conditioning coaches as it mitigates the need for 1RM tests. Also, PPO determination based on BM can be exercise specific, that is, learning and application of maximum tests in other weightlifting derivatives with higher complexity such as the WCD are unnecessary.

## Conclusion

The WPD have low technical complexity and may elicit similar or even greater power output than WCD (Comfort et al., 2011; Suchomel et al., 2014b; Suchomel and Sole, 2017). For this reason, it has been recommended the use of the WPD in training programs (Suchomel et al., 2015b, 2018). For strength and conditioning coaches who choose to use WPD at their respective PPO, using BM as a reference to determine the PPO can be a simple and practical alternative. This approach is less time-consuming and allows the determination of the optimal load in each specific exercise that will be included in the training program. However, for proper use of BM, it is necessary to carry out individual tests.

## Author Contributions

All authors contributed to the development of the manuscript, reviewed it, and approved the content of the final version.

## Conflict of Interest

The authors declare that the research was conducted in the absence of any commercial or financial relationships that could be construed as a potential conflict of interest.
